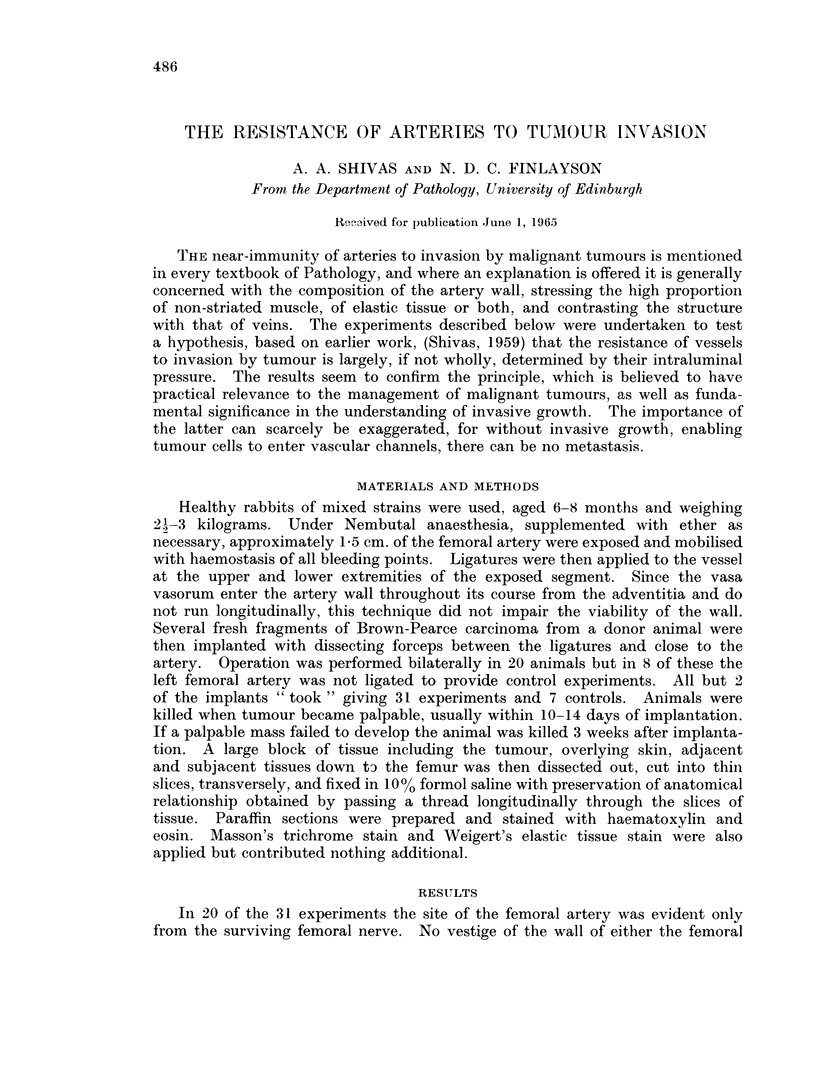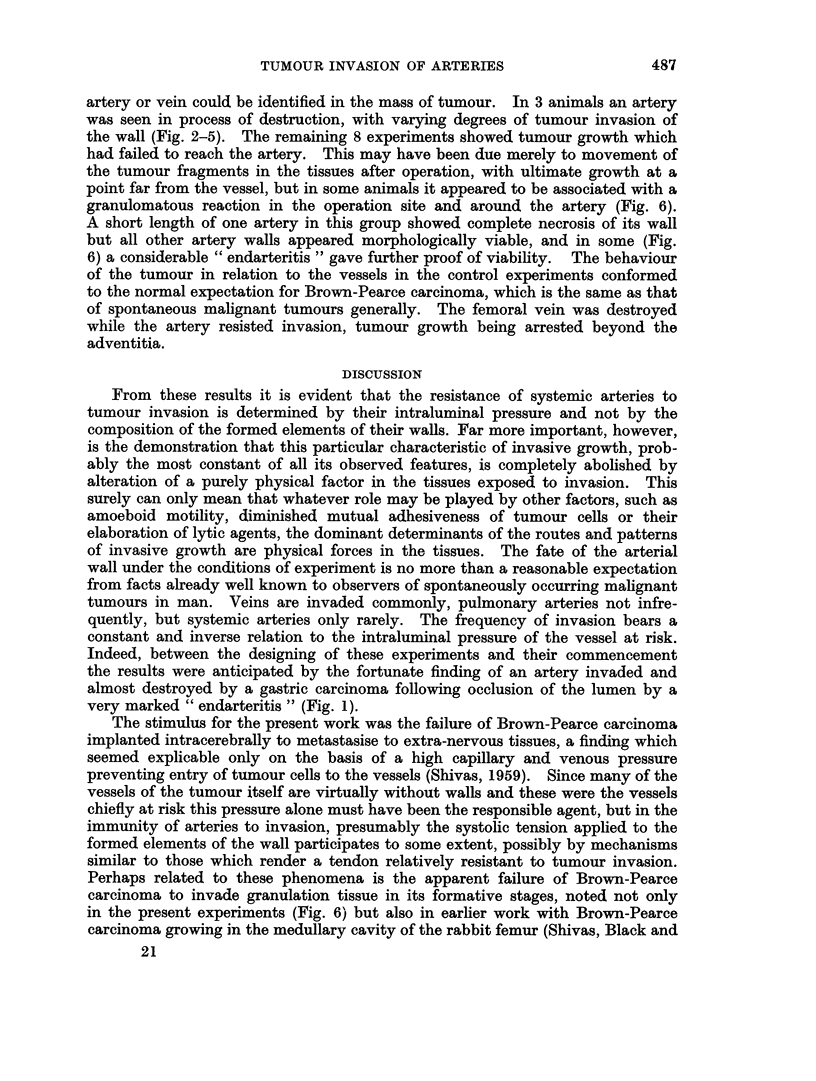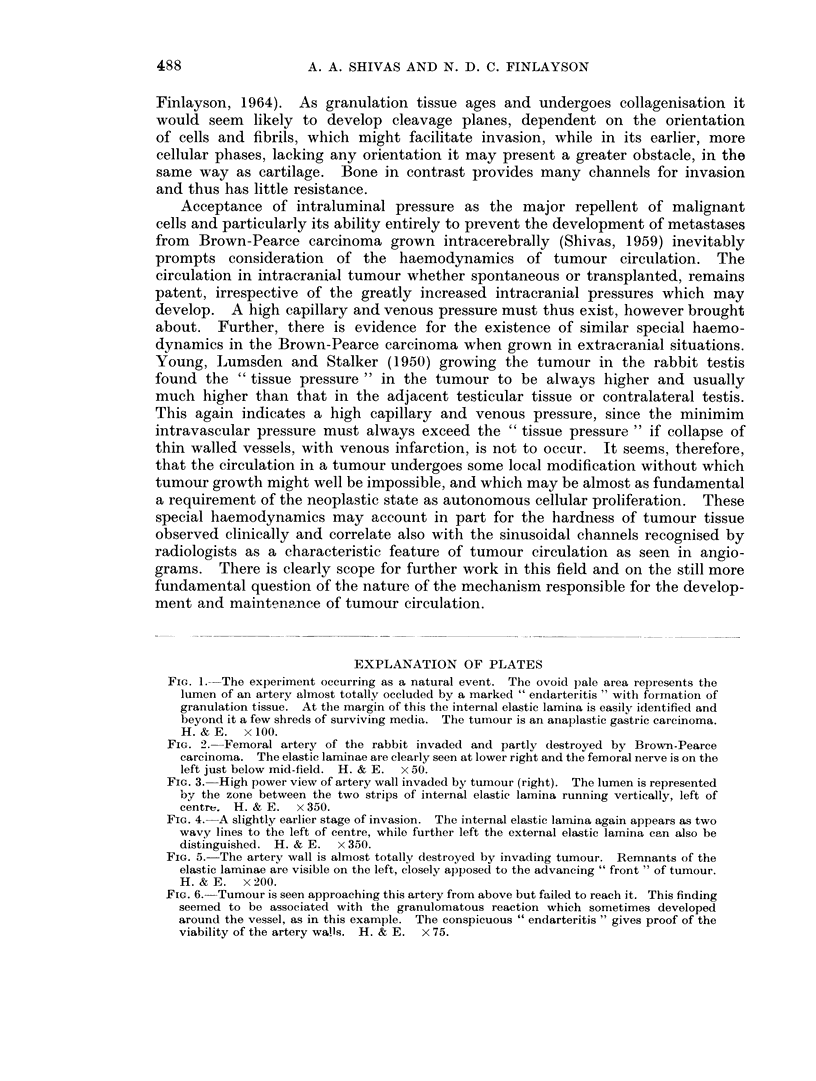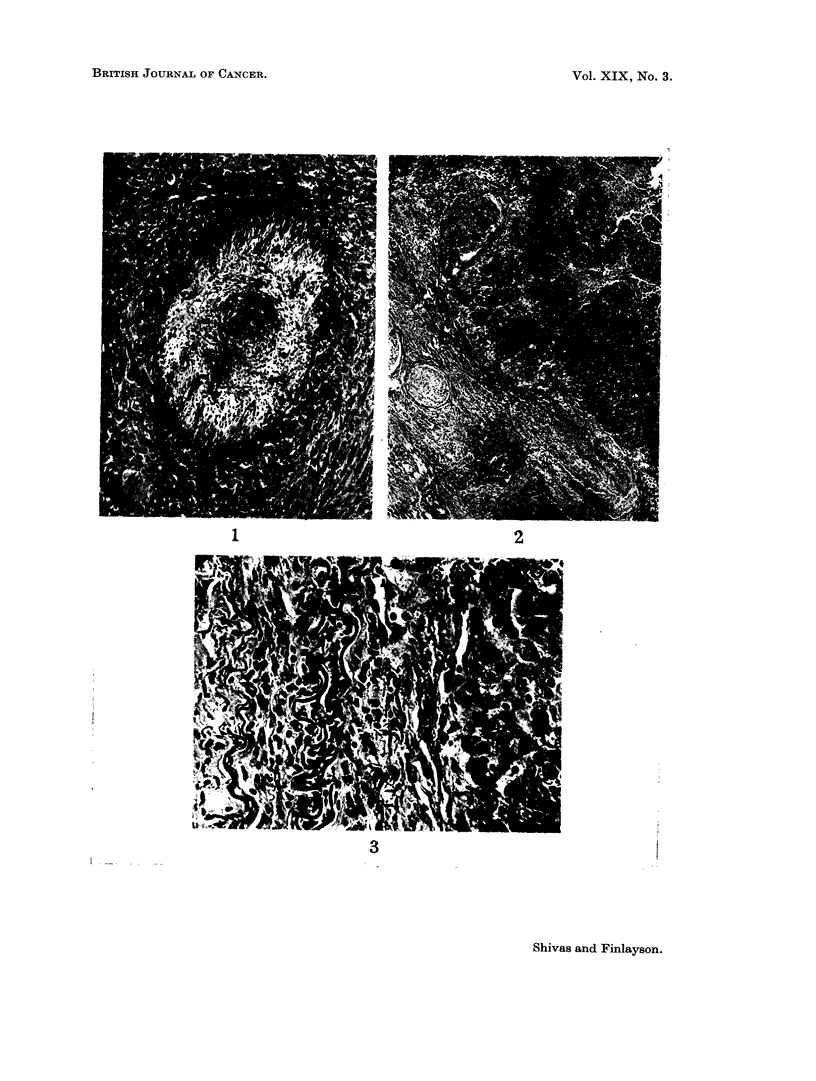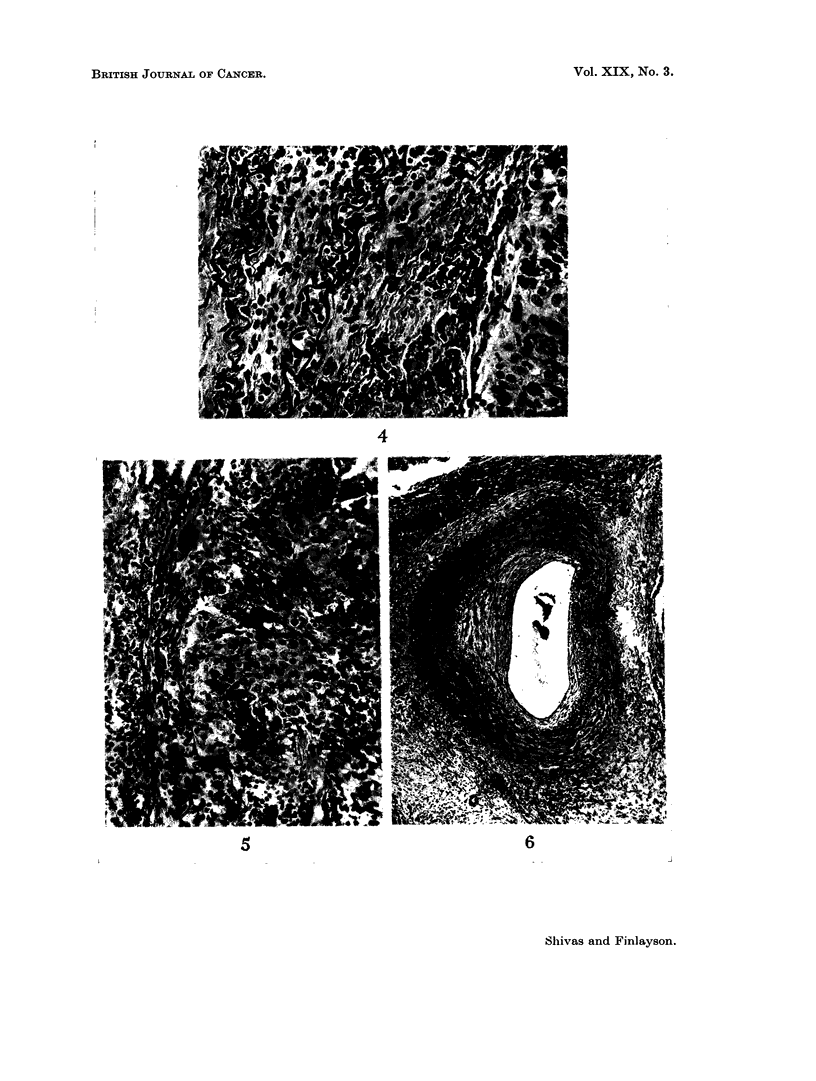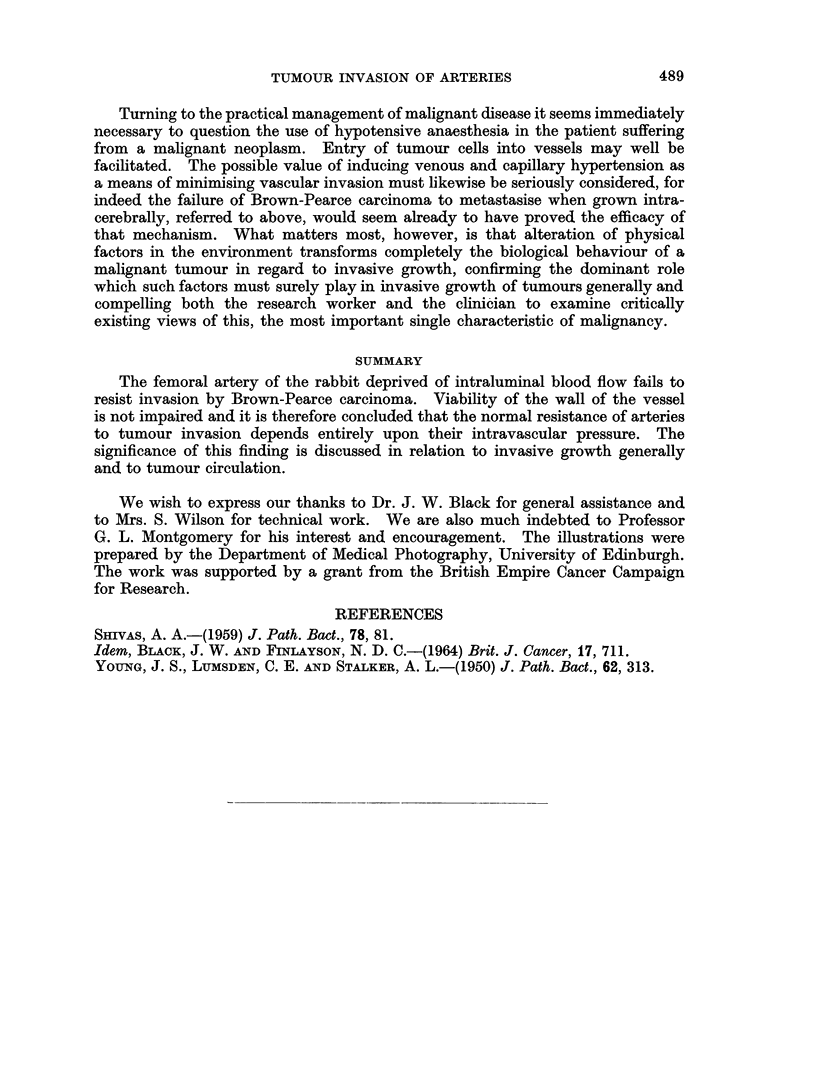# The resistance of arteries to tumour invasion.

**DOI:** 10.1038/bjc.1965.57

**Published:** 1965-09

**Authors:** A. A. Shivas, N. D. Finlayson

## Abstract

**Images:**


### THE RESISTANCE OF ARTERIES TO TUMOUR INVASION

A. A. SHIVAS AND N. D. C. FINLAYSON
*From the Department of Pathology, University of Edinburgh*

Received for publication June 1, 1965

THE near-immunity of arteries to invasion by malignant tumours is mentioned in every textbook of Pathology, and where an explanation is offered it is generally concerned with the composition of the artery wall, stressing the high proportion of non-striated muscle, of elastic tissue or both, and contrasting the structure with that of veins. The experiments described below were undertaken to test a hypothesis, based on earlier work, (Shivas, 1959) that the resistance of vessels to invasion by tumour is largely, if not wholly, determined by their intraluminal pressure. The results seem to confirm the principle, which is believed to have practical relevance to the management of malignant tumours, as well as fundamental significance in the understanding of invasive growth. The importance of the latter can scarcely be exaggerated, for without invasive growth, enabling tumour cells to enter vascular channels, there can be no metastasis.

### MATERIALS AND METHODS

Healthy rabbits of mixed strains were used, aged 6–8 months and weighing $2\frac{1}{2}$–3 kilograms. Under Nembutal anaesthesia, supplemented with ether as necessary, approximately 1·5 cm. of the femoral artery were exposed and mobilised with haemostasis of all bleeding points. Ligatures were then applied to the vessel at the upper and lower extremities of the exposed segment. Since the vasa vasorum enter the artery wall throughout its course from the adventitia and do not run longitudinally, this technique did not impair the viability of the wall. Several fresh fragments of Brown-Pearce carcinoma from a donor animal were then implanted with dissecting forceps between the ligatures and close to the artery. Operation was performed bilaterally in 20 animals but in 8 of these the left femoral artery was not ligated to provide control experiments. All but 2 of the implants " took " giving 31 experiments and 7 controls. Animals were killed when tumour became palpable, usually within 10–14 days of implantation. If a palpable mass failed to develop the animal was killed 3 weeks after implantation. A large block of tissue including the tumour, overlying skin, adjacent and subjacent tissues down to the femur was then dissected out, cut into thin slices, transversely, and fixed in 10% formol saline with preservation of anatomical relationship obtained by passing a thread longitudinally through the slices of tissue. Paraffin sections were prepared and stained with haematoxylin and eosin. Masson's trichrome stain and Weigert's elastic tissue stain were also applied but contributed nothing additional.

### RESULTS

In 20 of the 31 experiments the site of the femoral artery was evident only from the surviving femoral nerve. No vestige of the wall of either the femoral artery or vein could be identified in the mass of tumour. In 3 animals an artery was seen in process of destruction, with varying degrees of tumour invasion of the wall (Fig. 2–5). The remaining 8 experiments showed tumour growth which had failed to reach the artery. This may have been due merely to movement of the tumour fragments in the tissues after operation, with ultimate growth at a point far from the vessel, but in some animals it appeared to be associated with a granulomatous reaction in the operation site and around the artery (Fig. 6). A short length of one artery in this group showed complete necrosis of its wall but all other artery walls appeared morphologically viable, and in some (Fig. 6) a considerable " endarteritis " gave further proof of viability. The behaviour of the tumour in relation to the vessels in the control experiments conformed to the normal expectation for Brown-Pearce carcinoma, which is the same as that of spontaneous malignant tumours generally. The femoral vein was destroyed while the artery resisted invasion, tumour growth being arrested beyond the adventitia.

### DISCUSSION

From these results it is evident that the resistance of systemic arteries to tumour invasion is determined by their intraluminal pressure and not by the composition of the formed elements of their walls. Far more important, however, is the demonstration that this particular characteristic of invasive growth, probably the most constant of all its observed features, is completely abolished by alteration of a purely physical factor in the tissues exposed to invasion. This surely can only mean that whatever role may be played by other factors, such as amoeboid motility, diminished mutual adhesiveness of tumour cells or their elaboration of lytic agents, the dominant determinants of the routes and patterns of invasive growth are physical forces in the tissues. The fate of the arterial wall under the conditions of experiment is no more than a reasonable expectation from facts already well known to observers of spontaneously occurring malignant tumours in man. Veins are invaded commonly, pulmonary arteries not infrequently, but systemic arteries only rarely. The frequency of invasion bears a constant and inverse relation to the intraluminal pressure of the vessel at risk. Indeed, between the designing of these experiments and their commencement the results were anticipated by the fortunate finding of an artery invaded and almost destroyed by a gastric carcinoma following occlusion of the lumen by a very marked " endarteritis " (Fig. 1).

The stimulus for the present work was the failure of Brown-Pearce carcinoma implanted intracerebrally to metastasise to extra-nervous tissues, a finding which seemed explicable only on the basis of a high capillary and venous pressure preventing entry of tumour cells to the vessels (Shivas, 1959). Since many of the vessels of the tumour itself are virtually without walls and these were the vessels chiefly at risk this pressure alone must have been the responsible agent, but in the immunity of arteries to invasion, presumably the systolic tension applied to the formed elements of the wall participates to some extent, possibly by mechanisms similar to those which render a tendon relatively resistant to tumour invasion. Perhaps related to these phenomena is the apparent failure of Brown-Pearce carcinoma to invade granulation tissue in its formative stages, noted not only in the present experiments (Fig. 6) but also in earlier work with Brown-Pearce carcinoma growing in the medullary cavity of the rabbit femur (Shivas, Black and

Finlayson, 1964). As granulation tissue ages and undergoes collagenisation it would seem likely to develop cleavage planes, dependent on the orientation of cells and fibrils, which might facilitate invasion, while in its earlier, more cellular phases, lacking any orientation it may present a greater obstacle, in the same way as cartilage. Bone in contrast provides many channels for invasion and thus has little resistance.

Acceptance of intraluminal pressure as the major repellent of malignant cells and particularly its ability entirely to prevent the development of metastases from Brown-Pearce carcinoma grown intracerebrally (Shivas, 1959) inevitably prompts consideration of the haemodynamics of tumour circulation. The circulation in intracranial tumour whether spontaneous or transplanted, remains patent, irrespective of the greatly increased intracranial pressures which may develop. A high capillary and venous pressure must thus exist, however brought about. Further, there is evidence for the existence of similar special haemodynamics in the Brown-Pearce carcinoma when grown in extracranial situations. Young, Lumsden and Stalker (1950) growing the tumour in the rabbit testis found the " tissue pressure " in the tumour to be always higher and usually much higher than that in the adjacent testicular tissue or contralateral testis. This again indicates a high capillary and venous pressure, since the minimim intravascular pressure must always exceed the " tissue pressure " if collapse of thin walled vessels, with venous infarction, is not to occur. It seems, therefore, that the circulation in a tumour undergoes some local modification without which tumour growth might well be impossible, and which may be almost as fundamental a requirement of the neoplastic state as autonomous cellular proliferation. These special haemodynamics may account in part for the hardness of tumour tissue observed clinically and correlate also with the sinusoidal channels recognised by radiologists as a characteristic feature of tumour circulation as seen in angiograms. There is clearly scope for further work in this field and on the still more fundamental question of the nature of the mechanism responsible for the development and maintenance of tumour circulation.

---

### EXPLANATION OF PLATES

FIG. 1.—The experiment occurring as a natural event. The ovoid pale area represents the lumen of an artery almost totally occluded by a marked " endarteritis " with formation of granulation tissue. At the margin of this the internal elastic lamina is easily identified and beyond it a few shreds of surviving media. The tumour is an anaplastic gastric carcinoma. H. & E. ×100.

FIG. 2.—Femoral artery of the rabbit invaded and partly destroyed by Brown-Pearce carcinoma. The elastic laminae are clearly seen at lower right and the femoral nerve is on the left just below mid-field. H. & E. ×50.

FIG. 3.—High power view of artery wall invaded by tumour (right). The lumen is represented by the zone between the two strips of internal elastic lamina running vertically, left of centre. H. & E. ×350.

FIG. 4.—A slightly earlier stage of invasion. The internal elastic lamina again appears as two wavy lines to the left of centre, while further left the external elastic lamina can also be distinguished. H. & E. ×350.

FIG. 5.—The artery wall is almost totally destroyed by invading tumour. Remnants of the elastic laminae are visible on the left, closely apposed to the advancing " front " of tumour. H. & E. ×200.

FIG. 6.—Tumour is seen approaching this artery from above but failed to reach it. This finding seemed to be associated with the granulomatous reaction which sometimes developed around the vessel, as in this example. The conspicuous " endarteritis " gives proof of the viability of the artery walls. H. & E. ×75.

1

2

3

Shivas and Finlayson.

4

5

6

Turning to the practical management of malignant disease it seems immediately necessary to question the use of hypotensive anaesthesia in the patient suffering from a malignant neoplasm. Entry of tumour cells into vessels may well be facilitated. The possible value of inducing venous and capillary hypertension as a means of minimising vascular invasion must likewise be seriously considered, for indeed the failure of Brown-Pearce carcinoma to metastasise when grown intracerebrally, referred to above, would seem already to have proved the efficacy of that mechanism. What matters most, however, is that alteration of physical factors in the environment transforms completely the biological behaviour of a malignant tumour in regard to invasive growth, confirming the dominant role which such factors must surely play in invasive growth of tumours generally and compelling both the research worker and the clinician to examine critically existing views of this, the most important single characteristic of malignancy.

### SUMMARY

The femoral artery of the rabbit deprived of intraluminal blood flow fails to resist invasion by Brown-Pearce carcinoma. Viability of the wall of the vessel is not impaired and it is therefore concluded that the normal resistance of arteries to tumour invasion depends entirely upon their intravascular pressure. The significance of this finding is discussed in relation to invasive growth generally and to tumour circulation.

We wish to express our thanks to Dr. J. W. Black for general assistance and to Mrs. S. Wilson for technical work. We are also much indebted to Professor G. L. Montgomery for his interest and encouragement. The illustrations were prepared by the Department of Medical Photography, University of Edinburgh. The work was supported by a grant from the British Empire Cancer Campaign for Research.

### REFERENCES

Shivas, A. A.—(1959) *J. Path. Bact.*, **78**, 81.
*Idem*, Black, J. W. and Finlayson, N. D. C.—(1964) *Brit. J. Cancer*, **17**, 711.
Young, J. S., Lumsden, C. E. and Stalker, A. L.—(1950) *J. Path. Bact.*, **62**, 313.